# New Anæsthetic Agent—Kerosolene

**Published:** 1861-10

**Authors:** 


### EDITORIAL DEPARTMENT.

### NEW ANÆSTHETIC AGENT—KEROSOLENE.

Dr. Bowditch presented several bottles containing a liquid alleged to possess anæsthetic properties, and read the following letter from Mr. Joshua Merrill, of South Boston, describing it:—

"Gentlemen,—The article I now present for your consideration is from a class of volatile hydro-carbons derived from the decomposition of coal at low temperatures, which decomposition produces *liquid* products instead of prominent *gases*. The article before you is the most volatile of the liquids, carefully separated by distillation, an dthen purified by treating it with those chemicals which best remove all foreign organic matter from it. It is, I believe, the lightest, specifically, of all known liquids, its specific gravity being from 615 to 635, water being 1000 (ether being from 713 to 715.) It is, I believe, chemically a pure liquid hydro-carbon. Just its equivalents of carbon and hydrogen I am unable to give; in fact, I believe it has never been subjected to analysis. I have presented this sample with the view of interesting the medical gentlemen connected with the Society, in its peculiar property of producing anæsthesia. My attention was first called to its anæsthetic power, by some of the workmen employed in its manufacture becoming partially or wholly insensible from inhaling its vapors. I have personally often been under its influence in a small degree—enough to produce peculiar lightness of the head, and weakness of the limbs. In all cases which have come under my observation, those persons who have inhaled it rapidly recovered when brought to the open air; in from ten to fifteen minutes perfect restoration ensues, and the men are able to resume their employment. It is, like ether, explosive when its vapors are largely mixed with atmospheric air; the liquid readily ignites, rendering care necessary when artificial light is used during experiments. Its cost is comparatively small—one dollar per gallon will, I think, furnish it in quantities. In conclusion, if I have brought to your notice a cheap and *safe* anæsthetic agent, my object has been accomplished, leaving to the Society those experiments necessary to establish its value, or otherwise.

I am, respectfully yours, Joshua Merrill, *Sup't D. K. O. Co.*"

Dr. H. J. Bigelow remarked, that in reference to any new anæsthetic, several points required consideration, and among these he would mention some in reference to the agent now submitted to the Society, and which he now saw for the first time. First, its efficiency ; of this he was satisfied by the few inhalations he had just made from the bottle. He believed it as strong, at least, as ether. Second, it is tasteless as water, while its vapor is in no way irritating. On the contrary, its flavor is agreeable, and resembles a dilute chloroform, with a whiff of coal tar or creosote. What is remarkable, this odor, which is quite evident as the fluid evaporates, leaves, when it is dry, not a trace, either of the chloric ether smell or of the creosote, being in this respect wholly unlike ether or chloroform. It is also abundant and cheap. It remains to be settled whether this agent is productive of headache and other simptoms, like amylene, or whether it be, even in rare instances, fatal without warning, like chloroform. But this last is unlikely, inasmuch as the agent seems to be dilute, and not concentrated like chloroform. Nausea is, of course, a necessary accompaniment of certain cerebral disturbance. If this is an anæsthetic of the character it seems to be, at once effectual, agreeable, tasteless, without subsequent flavor or odor, it will supercede ether ; and it has certainly, at this moment, a remarkable air of promise.

On motion of Dr. Bowditch, the new agent was referred to the Hospital Surgeons and Dr. Bacon for experiment, and on motion of Dr. Storer, Dr. Bigelow was requested to draw up a report on the results of their investigations.—[*Boston Medical Journal, Aug.* 22.

